# Extended-release metformin improves cognitive impairment in frail older women with hypertension and diabetes: preliminary results from the LEOPARDESS Study

**DOI:** 10.1186/s12933-023-01817-4

**Published:** 2023-04-21

**Authors:** Pasquale Mone, Giuseppe Martinelli, Angela Lucariello, Anna Luisa Leo, Anna Marro, Stefano De Gennaro, Stefania Marzocco, Divina Moriello, Salvatore Frullone, Luigi Cobellis, Gaetano Santulli

**Affiliations:** 1 Department of Medicine, Division of Cardiology, Einstein Institute for Aging Research, Fleischer Institute for Diabetes Research (FIDAM), Einstein - Mount Sinai Diabetes Research Center (ES-DRC), Albert Einstein College of Medicine, New York City, NY USA; 2 ASL Avellino, Avellino, Italy; 3 University of Campania “Luigi Vanvitelli”, Caserta, Italy; 4 Parthenope University Naples, Naples, Italy; 5 Department of Pharmacy, University of Salerno, Fisciano, Italy; 6 Department of Molecular Pharmacology, Institute for Neuroimmunology and Inflammation (INI), Albert Einstein College of Medicine, New York City, NY USA

**Keywords:** Aging, Biguanides, Cognitive dysfunction, Frailty, Hyperglycemia, Menopause, Metformin, Sex differences, Women’s health

## Abstract

**Background:**

Women have a high risk of frailty independently of age and menopause state. Diabetes and hypertension increase the risk of frailty and cognitive impairment. Metformin has been employed in post-menopausal women and some reports have shown encouraging effects in terms of attenuated frailty. However, the impact on cognitive performance of a recently introduced extended-release formulation of metformin has never been explored.

**Methods:**

We studied consecutive frail hypertensive and diabetic older women presenting at the ASL (local health authority of the Italian Ministry of Health) Avellino, Italy, from June 2021 to August 2022, who were treated or not with extended-release metformin. We included a control group of frail older males with diabetes and hypertension treated with extended-release metformin and a control group of frail older women with diabetes and hypertension treated with regular metformin.

**Results:**

A total of 145 patients successfully completed the study. At the end of the 6-month follow-up, we observed a significantly different cognitive performance compared to baseline in the group of frail women treated with extended-release metformin (p: 0.007). Then, we compared the follow-up groups and we observed significant differences between frail women treated vs. untreated (p: 0.041), between treated frail women and treated frail men (p: 0.016), and between women treated with extended-release metformin vs. women treated with regular metformin (p: 0.048). We confirmed the crucial role of extended-release metformin applying a multivariable logistic analysis to adjust for potential confounders.

**Conclusions:**

We evidenced, for the first time to the best of our knowledge, the favorable effects on cognitive impairment of extended-release metformin in frail women with diabetes and hypertension.

## Background

Frailty is a multidimensional condition typical of older adults driving worst outcomes including cognitive and functional decline, hospitalization, and death [1–4]. We and others have previously shown the association between frailty and hypertension [5–11]. Equally important, diabetes is a condition extremely common in frailty and represents a distinctive determinant of cognitive decline [12–14]. In a recent report, our group highlighted a strong correlation between physical and cognitive impairment in frail older adults [15].

Metformin is an oral antidiabetic drug that remains a cornerstone of the first-line therapeutic approach in both elder and younger populations [16–19]. Interestingly, recent studies have also suggested beneficial effects in post-menopausal women [20–22] and some reports have shown advantageous actions in terms of attenuated parameters of frailty [23–25].

The recently introduced extended-release formulation of metformin has shown less gastrointestinal effects compared to the regular (immediate-release) metformin and this aspect might be critical in elders, which are often overtreated [26–32]. Previous studies have reported differences in cognitive function when comparing extended-release to regular formulations [33–36]. However, to our knowledge the effects of extended-release metformin have never been assessed in frail older patients with diabetes and hypertension.

Women have a higher risk of frailty during lifespan [37] and previous investigations have evidenced that this aspect is independent of age and menopause state [38, 39]. Thus, also based on its increased tolerability and decreased side effects [40, 41] we hypothesized that extended-release metformin may be helpful in frail women with diabetes and hypertension. On these grounds, we investigated the effects of extended-release metformin on cognitive performance in this population.

## Methods

We designed the LEOPARDESS (extended-reLease mEtformin and cOgnitive imPAirment in fRail olDer womEn with hypertenSion and diabeteS) study, enrolling consecutive frail hypertensive older women with diabetes presenting at the ASL (local health authority of the Italian Ministry of Health) Avellino, Italy, from June 2021 to August 2022. As a control population, we evaluated an age-matched group of frail hypertensive older males with diabetes and hypertension treated with extended-release metformin, a formulation of metformin hydrochloride suspended within a polymer matrix that dissolves over hours as the tablet passes through the gastrointestinal tract.

Inclusion criteria:

1. Diagnosis of diabetes;
2. Diagnosis of hypertension with no clinical or laboratory evidence of secondary causes;
3. Age > 65 years;
4. Montreal Cognitive Assessment (MoCA) Score < 26.
5. Diagnosis of frailty.

 Exclusion criteria:

1. Age ≤ 65 years;
2. Absence of frailty;
3. MoCA score ≥ 26;
4. Absence of diabetes and hypertension;
5. Glomerular Filtration Rate (GFR) < 30.

Diabetes was defined according to the guidelines of the American Diabetes Association (ADA) [42]. Hypertension was defined as systolic blood pressure (SBP) ≥ 140 mmHg and/or diastolic blood pressure (DBP) ≥ 90 mmHg on repeated measurements, or use of antihypertensive medications [43, 44]. All patients had blood samples drawn for measuring glycemia, HbA1c, and creatinine. The study was approved by the local Ethical Committee (Campania Nord). All subjects signed an informed consent.

### Frailty assessment

As previously described [12, 45], we diagnosed frailty when at least three of the following five criteria were present:

- Weight loss (unintentional loss ≥ 4.5 kg in the past year).
- Weakness (handgrip strength in the lowest 20% quintile at baseline, adjusted for sex and body mass index, BMI).
- Exhaustion (poor endurance and energy, self-reported).
- Slowness (walking speed under the lowest quintile adjusted for sex and height).
- Low physical activity level (lowest quintile of kilocalories of physical activity during the past week).

### Global cognitive evaluation

We screened global cognitive function using the MoCA Test, a cognitive test largely considered the best option to diagnose mild cognitive impairment [46]. We defined cognitive impairment by cut-off values < 26, as specified in the inclusion criteria [47, 48].

### Extended-release metformin treatment

Our population was divided in three different groups: patients receiving 500 mg extended-release metformin, patients receiving 500 mg regular metformin, and patients not receiving metformin; we also examined a control group of age-matched frail older males with diabetes and hypertension, receiving 500 mg extended-release metformin. All patients were followed-up for six months.

### Statistical analysis

Data are presented as means ± SD. Based on our preliminary findings, we had calculated the number of patients required for the study to have 95% power with a two-tailed type I error at the 0.05 level of significance; the minimum sample size was predicted using G_POWER software, yielding an estimated sample of 90 patients. Multivariable regression models fitted to assess the association between cognitive impairment and covariates. All calculations were performed using SPSS 26 (IBM, Armonk, NY) and GraphPad Prism 9.0 (San Diego, CA).

## Results

**Fig. 1:**
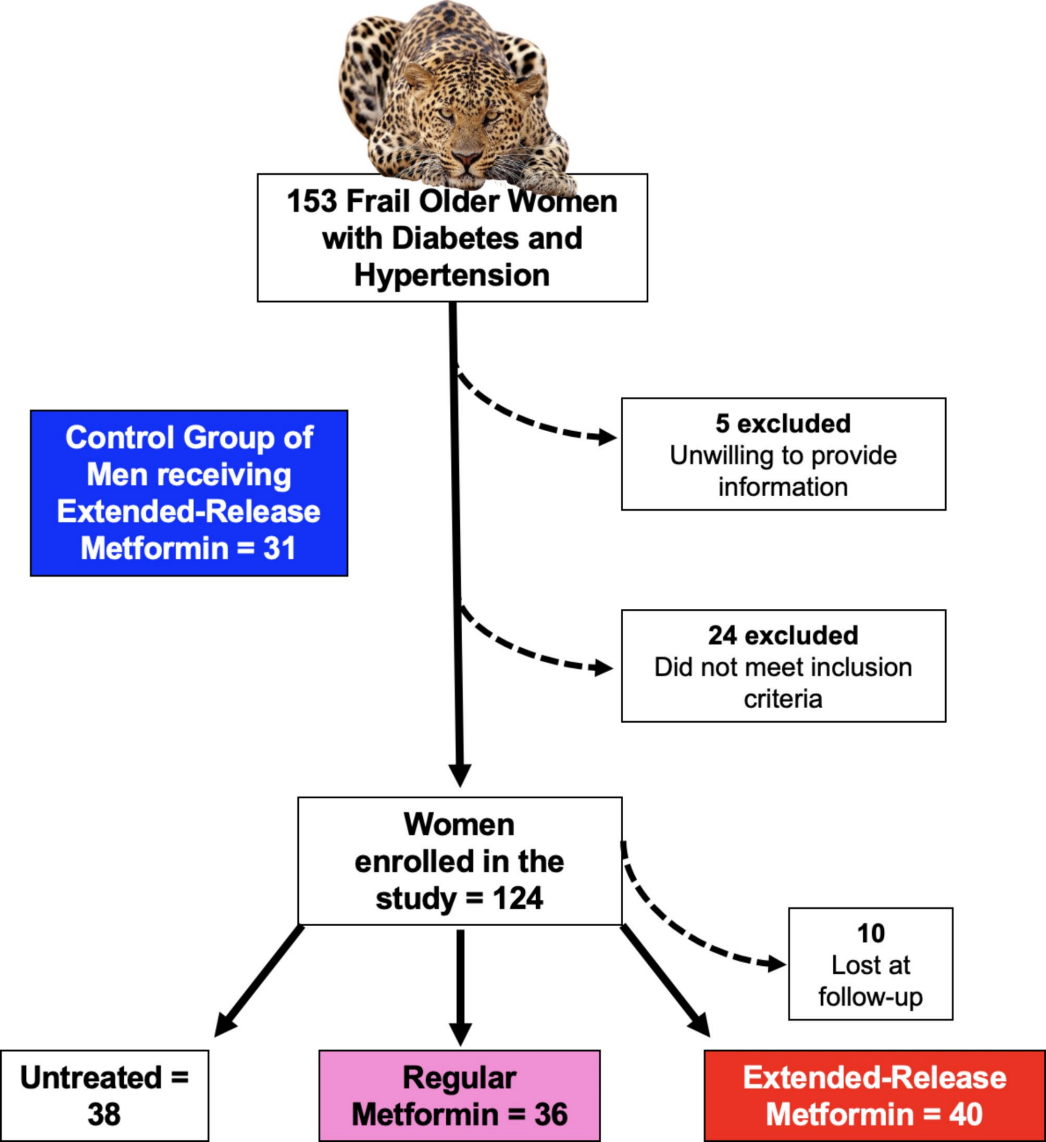
Flow-chart of the LEOPARDESS (extended-reLease mEtformin and cOgnitive imPAirment in fRail olDer womEn with hypertenSion and diabeteS) study

We screened 153 consecutive cognitively impaired frail older women with diabetes and hypertension. We excluded 29 women because they did not meet the afore-mentioned inclusion criteria. Hence, we enrolled 124 women. Of these, 40 were assigned to be treated with extended-release metformin, 36 received regular metformin, and 38 did not receive metformin (Fig. 1). At the end of the 6-month follow-up, data from a total of 10 women were not available (4 from the untreated group, and 3 each from the groups receiving regular or extended-release metformin); so, 114 women successfully completed the study. We also examined a control group of age-matched 31 frail older males with diabetes and hypertension who received extended-release metformin. The baseline characteristics of these patients are reported in Table 1.

**Table 1:** Baseline characteristics of our patients. Data are means ± SD or n (%). BMI: body mass index. SBP: systolic blood pressure. DBP: diastolic blood pressure. HR: Heart rate. COPD: chronic obstructive pulmonary disease. CKD: chronic kidney disease. HDL: High-density lipoprotein; LDL: Low-density lipoprotein. HbA1c: glycated hemoglobin.

	Extended-release Metformin Women	Non-Treated Women	Regular Metformin Women	Extended-release Metformin Men
N	40	38	36	31
Age (years)	77.1 ± 7.8	75.6 ± 5.5	76.0 ± 5.2	75.7 ± 6.0
BMI (kg/m^2^)	29.5 ± 3.4	28.6 ± 3.2	29.6 ± 3.6	28.5 ± 3.3
SBP (mmHg)	132.8 ± 10.8	133.1 ± 12.1	131.8 ± 9.5	133.4 ± 11.5
DBP (mmHg)	81.8 ± 9.9	81.5 ± 10.2	81.6 ± 9.0	82.0 ± 10.4
HR (bpm)	73.8 ± 9.0	73.1 ± 9.0	74.5 ± 9.4	73.5 ± 8.6
***Comorbidities***				
Dyslipidemia, n (%)	30 (75.0)	27 (72.0)	26 (73)	22 (71.0)
COPD, n (%)	14 (35.0)	13 (34.0)	13 (36)	12 (39.0)
CKD, n (%)	15 (37.0)	15 (39.0)	14 (39)	12 (39.0)
***Laboratory parameters***				
Plasma glucose (mg/dl)	160.8 ± 49.1	155.3 ± 39.4	155.5 ± 40.6	156.6 ± 40.4
Cholesterol (mg/dl)	195.2 ± 18.2	194.9 ± 18.7	193.1 ± 18.8	196.0 ± 19.1
LDL-cholesterol (mg/dl)	130.3 ± 16.7	130.0 ± 16.5	129.6 ± 16.4	130.5 ± 16.8
HDL-cholesterol (mg/dl)	39.5 ± 3.6	38.9 ± 3.7	38.7 ± 3.9	39.1 ± 3.4
Creatinine (mg/dl)	1.0 ± 0.2	0.9 ± 0.1	0.9 ± 0.2	0.9 ± 0.2
HbA1c (%)	7.2 ± 0.6	7.1 ± 0.7	7.1 ± 0.7	7.4 ± 0.5

**Fig. 2:**
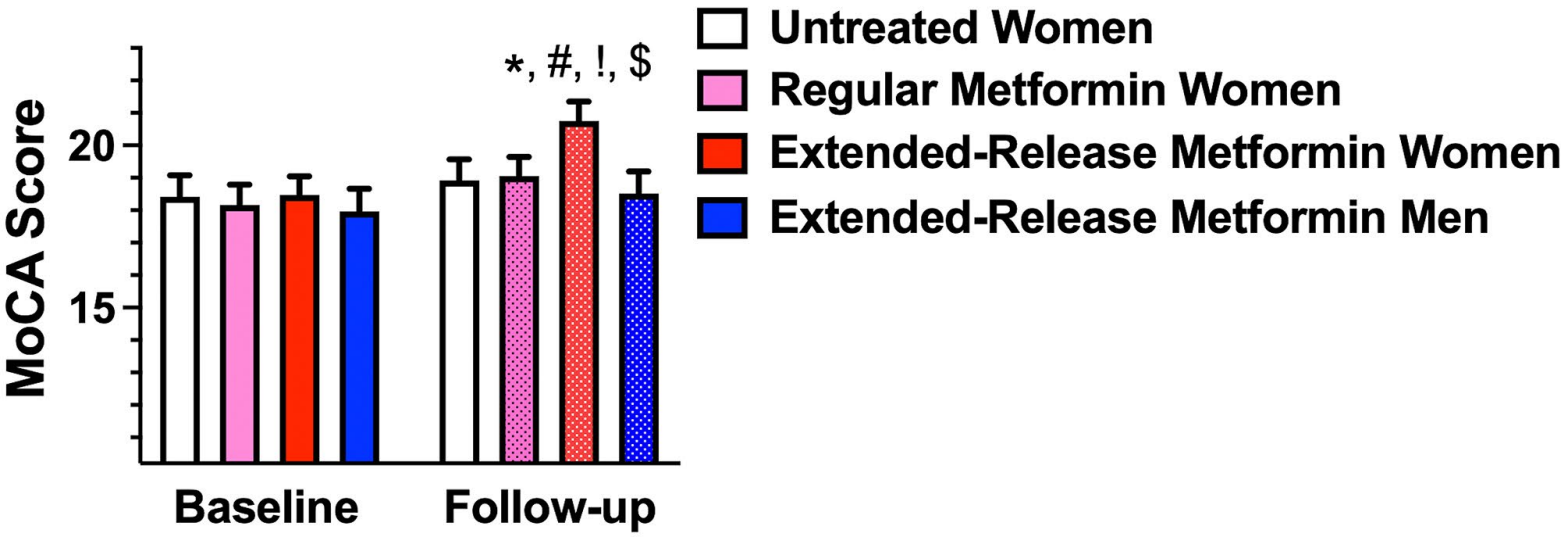
Evaluation of cognitive dysfunction at baseline and at 6-month follow-up in the indicated groups of patients. Data are mean±SD; *: p < 0.05 vs. baseline; #: p < 0.05 vs. untreated; !: p < 0.05 vs. regular metformin, $: p < 0.05 vs. extended-release metformin men.

At the end of the 6-month follow-up, we observed a significant (p: 0.007) difference compared to baseline in the frail women group treated with extended-release metformin (Fig. 2). Remarkably, we found no significant difference between baseline and follow-up in the other groups.

Then, we compared the follow-up groups to verify the presence of differences among groups. We found a significant difference at follow-up between frail women treated with extended-release metformin vs. non-treated (p: 0.041), between treated frail women vs. treated frail men (p: 0.016), and between women treated with extended-release metformin vs. women treated with regular metformin (p: 0.048).

We confirmed the critical role of extended-release metformin by a multivariable logistic analysis after adjusting for potential confounders (Table 2), including age, BMI, systolic and diastolic blood pressure, heart rate, blood glucose, and serum creatinine; these covariates were selected because they have been previously associated with cognitive impairment [7, 49–64].

**Table 2:** Multivariable analysis with MoCA score as the dependent variable

				95.0% Confidence Interval for B	
	B	Standard Error	p	Lower Bound	Upper Bound
Age	-0.218	0.053	< 0.001	-0.324	-0.113
BMI	-0.303	0.090	0.001	-0.481	-0.126
Glucose	-0.008	0.008	0.292	-0.024	0.007
Creatinine	2.576	1.605	0.111	-0.599	5.751
SBP	-0.066	0.049	0.184	-0.163	0.032
DBP	0.050	0.062	0.420	-0.073	0.174
HR	0.000	0.043	0.996	-0.085	0.085
Extended-Release Metformin	2.389	0.639	< 0.001	1.125	3.652

BMI: body mass index. SBP: systolic blood pressure. DBP: diastolic blood pressure. HR: Heart rate

## Discussion

In the present study we have demonstrated, for the first time to the best of our knowledge, the favorable effects of extended-release metformin on cognitive impairment in frail women with diabetes and hypertension.

Sex differences are pivotal in the management of frailty and cognitive impairment. In this context, extended-release metformin may play a decisive role in reducing cognitive impairment in frail women with hypertension and diabetes. Many drugs have been investigated in post-menopausal women [65–67] and metformin has been proposed as an anti-aging drug [68, 69]. Intriguingly, preclinical assays in rodents had evidenced differences in the effects of metformin on lifespan [70, 71]. We conjectured that the extended-release formulation could be decisive in reducing the adverse effects of metformin in frail women. Indeed, gastroenteric effects of normal metformin are often key determinants in discontinuing therapy. Additionally, metformin has been shown to have pleiotropic effects in women with polycystic ovary syndrome [72] and in preventing breast cancer [73, 74]. On these grounds, we believe that extended-release metformin exerted beneficial effects in our population because of its safe profile and multifactorial functions. We can only speculate on the potential molecular mechanisms underlying the observed phenotype, especially considering that although metformin and other biguanides are known to work by inhibiting hepatic gluconeogenesis, its precise mechanisms of action remain widely debated. Nir Barzilai and collaborators have shown that 6 weeks of metformin can improve age-associated metabolic derangements in glucose intolerant older adults: the treatment had tissue‐specific effects on gene expression and influenced not only metabolic genes and pathways, but also collagen and mitochondrial genes in adipose tissue, and DNA repair genes in skeletal muscle, emphasizing its targeting of multiple hallmarks of aging [75]. Most recently, researchers from Stanford University [76] have examined the genome-wide DNA methylation profile of metformin users, concluding that epigenetic modifications may underlie the previously reported anti-aging role of this drug. Metformin has been shown to affect the receptors for adiponectin, insulin, and several cytokines, and, all pathways that when inhibited are associated with longevity; at the intracellular level, biguanids inhibit inflammatory pathways and activate AMPK, thereby inhibiting mTOR, a major target in aging-related processes [77, 78]. Metformin also regulates protein synthesis, stress defense, mitochondrial function, oxidative stress, and autophagy, phenomena that are known to be associated with aging/longevity [79, 80]. Henceforth, a sustained action of metformin achieved via the extended-release formulation could be affecting any of the above-mentioned mechanisms.

Our results are noteworthy but not exempts of limitations, including the relatively small sample size and the brief follow-up. However, our population was a real-world homogeneous cluster and we had performed an a priori power analysis; additionally, observing effects already at 6-months could be seen as a *plus*. Nevertheless, further studies in large populations and with a longer follow-up are warranted to confirm our results. Moreover, since our study was performed in medical centers and health units that mostly see White patients, our findings may not be applicable to subjects with a different ethnical background.

## Conclusions

We evidenced the favorable effects on cognitive impairment of extended-release metformin in frail women with diabetes and hypertension.

## Data Availability

All data and materials are available from the first author upon reasonable request.
